# Properties of essential oils absorbed on the surface of cardboard pieces after using atmospheric-pressure plasma treatments to develop long-lasting *Varroa* miticides in honeybees (*Apis mellifera*)

**DOI:** 10.1371/journal.pone.0297980

**Published:** 2024-02-08

**Authors:** Thummanoon Boonmee, Chainarong Sinpoo, Laedlugkana Wongthaveethong, Terd Disayathanoowat, Pradoong Suanpoot, Jeffery S. Pettis, Veeranan Chaimanee

**Affiliations:** 1 Department of Agro-Industrial Biotechnology, Maejo University Phrae Campus, Phrae, Thailand; 2 Bee Protection Laboratory, Department of Biology, Faculty of Science, Chiang Mai University, Chiang Mai, Thailand; 3 Office of Research Administration, Chiang Mai University, Chiang Mai, Thailand; 4 Research Center of Deep Technology in Beekeeping and Bee Products for Sustainable Development Goals (SMART BEE SDGs), Chiang Mai University, Chiang Mai, Thailand; 5 Department of Forest Industry Technology, Maejo University Phrae Campus, Phrae, Thailand; 6 Pettis and Assoc. LLC, Salisbury, Maryland, United States of America; University of Alberta, CANADA

## Abstract

The ectoparasitic mite, *Varroa destructor* is the most serious widespread pest of managed honeybees (*Apis mellifera*). Several acaricide products, which include essential oils, have been proposed for mite control. In this study, we aimed to apply atmospheric-pressure plasma to modify a cardboard piece surface in order to prolong the delivery of essential oils for controlling *Varroa* in honeybee colonies. Absorption capacity, release rates and evaporation rates of essential oils were determined. Cardboard piece showed a higher absorption capacity of cinnamon compared to citronella and clove. Surface modification of cardboard pieces using argon plasma at different gas flow rates and treatment durations, significantly affected the absorption of clove oil. Additionally, the release rate of cinnamon, citronella and clove was significantly enhanced after argon plasma treatments. Evaporation of cinnamon was dramatically increased by plasma treatment at 6-h of incubation. The highest evaporation rate was obtained by plasma-treated cardboard piece at a gas flow rate of 0.5 Lpm for 60 s (0.2175 ± 0.0148 μl/g•h). Efficiency of plasma-treated cardboard piece, impregnated with essential oils, was also investigated for *Varroa* control in honeybee colonies. In the first experiment, formic acid 65% (v/v) showed the highest efficiency of 90.60% and 81.59% with the percent of mite infestation was 0.23 ± 0.13% and 0.47 ± 0.19% at 21 and 35 days, respectively after treatment. The efficacy of cardamon oil (5% (v/v)) delivered using plasma-treated cardboard pieces was 57.71% (0.70 ± 0.16% of mite infestation) at day 21 of experiment. However, the delivery of cardamon oil at the concentration of 1% and 5% (v/v) by untreated cardboard piece had 16.93% and 24.05% of efficacy to control mites. In the 2^nd^ experiment, the application of plasma-treated cardboard pieces impregnated with 5% (v/v) clove oil induced a 38.10% reduction in the population of *Varroa* mites followed by 5% (v/v) of cardamon with 30% efficiency. Although, the infestation rate of *Varroa* in colonies was not significant different between treatments, essential oils delivered using plasma-treated cardboard pieces tended to decrease *Varroa* population in the treated colonies. Hence, atmospheric-pressure plasma for the modification of other materials, should be further investigated to provide alternative control treatment applications against honeybee mites.

## Introduction

The ectoparasitic mite, *Varroa destructor* Anderson and Trueman is the most serious widespread pest of managed honeybee (*Apis mellifera* L.) and causes colony losses worldwide [1]. *Varroa* mites feed primarily on the fat body of immature and adult bees [2]. The effect of *Varroa* parasitism on the physiology of honeybees has been described, such as a reduction in protein content, lipid synthesis and body weight, and impaired immune function and shortened lifespan [3–6]. In addition, many viruses including deformed wing virus (DWV) and acute bee paralysis virus (ABPV) can be transmitted by *Varroa* mite [7, 8]. Reproduction of *Varroa* is related to brood availability in the colony [1]. In recent years, the prevalence of *Varroa* in honeybee colonies has been increased by several environmental factors and bees’ genotype [9–12]. The differences in genotypes influence the individual honeybee’s behavioural traits such as hygenic behaviour and grooming behaviour that are involved in resistance to *Varroa* [13, 14]. For instance, Africanized bees showed higher resistance to *Varroa* than European bees [9]. Additionally, temperature and humidity can have effects on honeybee activities which are linked to colony development and performance [15, 16]. Since mite infestation depends on colony development these environmental factors also affect mite reproduction in colony [15].

Synthetic chemical acaricides are widely used to control *Varroa*, especially formamidines (amitraz), pyrethroids (tau-fluvalinate) and organophosphates (coumaphos) [17]. However, mite populations are becoming increasingly resistant to these chemicals and their effectiveness have been reduced [18–20]. Therefore, increased doses of these acaricides have been applied in colonies, leading to the chemical contamination in honeybee products such as beeswax and honey [21]. Although, organic acids such as formic acid and oxalic acid are increasingly used for *Varroa* control, the use of formic acid to control mites can be harmful to bees [1, 22, 23]. Oxalic acid can vary widely in its effectiveness in controlling *Varroa* under colony conditions [24–26]. In recent years, there has been an increased interest in the use of natural compounds, especially essential oils to control honeybee mites [27–30]. In addition to acaricidal activity, application of essential oils showed low toxicity to bees and the residues of essential oil in honey pose minimal risk to human health [30–33]. Although, essential oils have shown acaricidal activity on *Varroa* mites, these oils are not consistently effective in honeybee colonies [34]. The concentrations of essential oils, solvents and or the type of media materials could all affect the distribution of essential oils for controlling *Varroa* mite in colonies. Therefore, developing a better delivery method for continuous release, to achieve the highly effective acaricides, would be an important improvement for the beekeeping industry.

Plasma surface modification is an efficient and powerful technique for the improvement of materials surface properties [35–37]. Ionized particles such as electrons, ions, neutrals, excited species, and reactive species generated through the plasma can be used to modify the surface properties of materials [38, 39]. Additionally, atmospheric-pressure plasma, using argon as the working gas at different flow rates and treatment durations, has been used for the surface modification of cardboard and drawing board pieces to improve their properties for distribution of essential oil to control *Tropilaelaps* spp. mites [40]. The absorption capacity of cardamon essential oil on cardboard piece was decreased by argon plasma but the release rate was not affected. The etching effect of plasma could be involved in the surface modification of cardboard. The fractured and scaly nature of the surface observed after plasma treatment could be involved in the essential oil absorption capacity of cardboard. The infestation of *Tropilaelaps* mites in a field experiment was lower in the plasma-treated cardboard piece, impregnated with essential oil [40].

Therefore, in the present study, suitable solvents for essential oils were investigated. Cardboard pieces were treated with argon plasma under different conditions and the absorption capacity, release and evaporation rates of the essential oils were determined. Lastly, the most promising combinations were tested for their acaricidal activity to control *Varroa* mites was tested under field conditions.

## Materials and methods

### Essential oils and solvents

Essential oils of clove (*Syzygium aromaticum*) (CAS No. 8000-34-8/84961-50-2), cinnamon (*Cinnamomum* sp.) (product code PO128351, Batch No. 990133) and citronella (*Cymbopogon* sp.) (Batch No. 0000065697) were purchased from the Union Science Co., Ltd. (Chiang Mai, Thailand). The dried fruit of cardamon (*Amomum krervanh*) was purchased from a herbal store in Chiang Mai and was extracted using a hydro-distillation method [33]. Their chemical compositions are shown in Table 1. Liquid paraffin (Labchem, Ajax Finechem Pty Ltd.), canola and sunflower oil, coconut oil (Naturel, Lam Soon (Thailand) Public Company Limited) and rice bran oil (King, Thai Edible Oil Co., Ltd.) were used to test for suitable solvents for the essential oils.

**Table 1:** Chemical compositions of essential oils used in this study.

Plant species	Chemical compositions		Reference
	Compound	%	
*Amomum krervanh*	1,8-Cineole	77.17	[33]
	β-Pinene	8.43	
	α-Terpineol	4.58	
*Cinnamomum* sp.	Not specified		Certificate of analysis
*Cymbopogon* sp.	Citronellal	40.73	Certificate of analysis
*Syzygium aromaticum*	Eugenol	85.74	Certificate of analysis
	Eugenol acetate	0.13	

### Materials

Commercial cardboard (grey board) No. 24 with smooth surface on both sides and in 2.06 mm thickness (manufacturer was not specified) was obtained from a store in Phrae, Thailand (S1 Fig). The pieces consisted of 1.0 cm × 1.0 cm and 2.5 cm × 22.0 cm were cut for the laboratory and field experiments, respectively.

### Suitable solvent for cinnamon oil

Canola, sunflower, coconut and rice bran oils were tested as solvents and compared with liquid paraffin. Cardboard pieces of 1.0 cm × 1.0 cm were immersed in cinnamon oil for 24 h, then removed and wiped with filter paper to remove excess essential oil. Afterward, each impregnated cardboard piece was incubated in 50 ml of each solvent at room temperature for 72 h in sealed beakers. Six replicates were analyzed for each solvent. The mixed solutions were measured for absorbance at 300 nm using a spectrophotometer (ThermoFisher Scientific GENESYS 10S) at 4, 8, 24, 32, 48, 56 and 72 hrs. The essential oil release rates were presented as the concentration of essential oil released (μl/ml) calculated from the standard curve of known concentrations of essential oil and absorbance [41].

### Atmospheric-pressure plasma treatment

**Fig 1:**
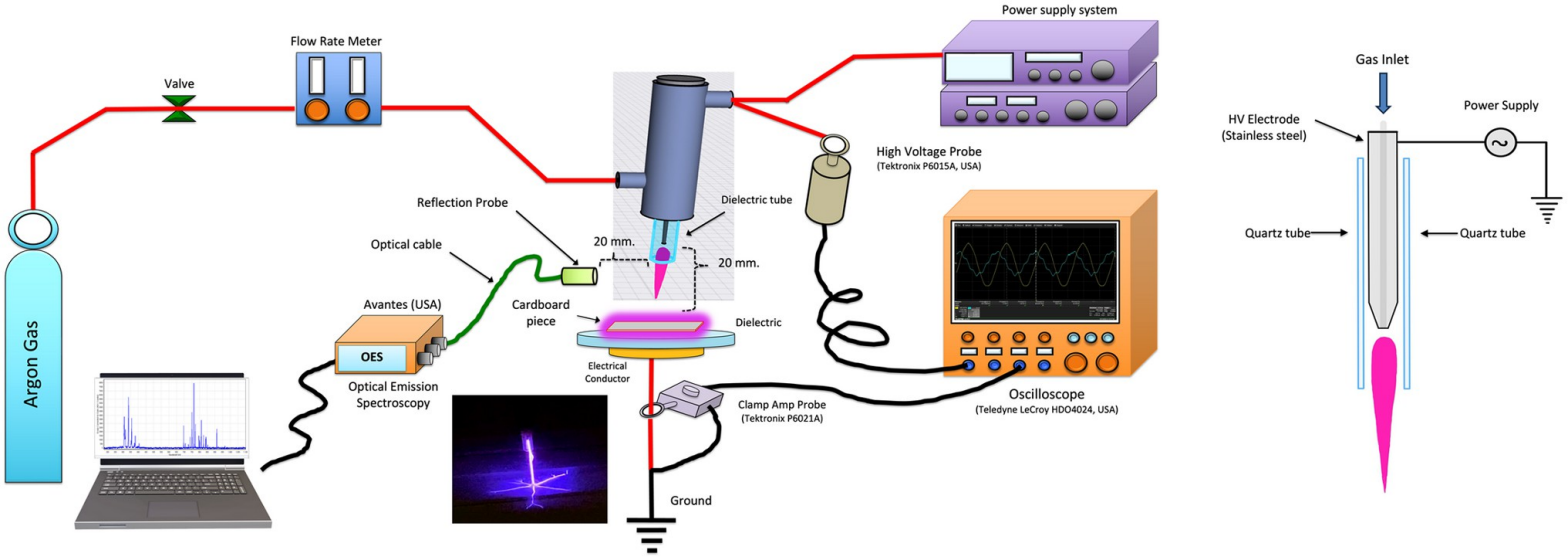
Schematic diagram of the atmospheric-pressure plasma jet system.

A schematic diagram of the atmospheric-pressure plasma jet system used in this study is shown in Fig 1. The plasma jet system is mainly composed of stainless steel electrodes, quartz dielectrics with an inner diameter of 4 mm and an outer diameter of 6 mm and a high-voltage power supply. The argon gas flow rate was monitored by a mass-flow controller. The electrical properties were measured by a high-voltage probe P6015A (Tektronix, USA). The voltage and current waveforms were recorded using oscilloscope HDO4024 (Teledyne LeCroy, USA) with a 200 MHz bandwidth and a 2.5 GS/s sampling rate. The emission spectra of plasma jet were determined in the wavelength range from 250 to 880 nm to identify the plasma constituent species by Optical Emission Spectroscopy (AVANTES, USA). Cardboard pieces were placed under atmospheric-pressure plasma jet nozzle with a working distance of 1.0 cm and run on both sides. Six atmospheric-pressure plasma conditions were applied at the gas flow rate of 0.25, 0.50 and 1.0 Litre per minute (Lpm) for 30 and 60 second. Untreated-cardboard pieces were included as positive control treatment. The replication in each treatment was 3–6 pieces. Cardboard pieces were further analyzed for absorption capacity, release rate and evaporation rate of essential oils.

### Essential oil absorption capacity, release rate and evaporation rate of cardboard pieces

The dry and impregnated (swollen) cardboard pieces were weighed using an analytical balance (accurate to 0.0001 g) (n = 3/treatment). The amount of absorbed essential oil was calculated based on the standard curve. The results were presented as μL of essential oil/mg of dry material [41]. Each swollen cardboard piece, after weighing, was incubated in 50 ml of liquid paraffin as dissolving agent (suitable solvent from the previous experiment) at room temperature for 72 h in sealed beakers. The release rate of essential oil was analyzed as described above. To determine the essential oil evaporation rate, cardboard pieces sized 1.0 cm × 1.0 cm were weighed before (W0) and after soaking (W1) in essential oil for 24 h. Each cardboard piece was then individually placed in a plastic container at 30° C and weighed at 2, 4, 6 and 8 h after incubation and every day for 14 days (W2). The evaporation level was calculated as: amount evaporated (W1-W2)/ amount absorbed (W1-W0) [42]. Then, the amount of evaporated essential oil at each time interval was presented as μL of essential oil/g of dry material/time (h) calculated based on the standard curve of known volume of essential oil and weight.

### Plasma-treated cardboard pieces impregnated with essential oil treatments for the control of *Varroa* mites under field conditions

No specific permits were required for the described field studies. The owner of the apiaries gave permission to conduct the study in the honeybee colonies. The field experiments were carried out in apiaries located in Salisbury Maryland, USA during August to September for the 1^st^ experiment and in October for the 2^nd^ experiment in 2021. A total of fifty honeybee colonies which included one brood chamber of 10 Langstroth frames, a queen excluder and a honey super, with adult bees covering 6–10 frames were used for the first experiment (S2 Fig). Before initializing the experiments, colonies were evaluated for adult bees and mite populations. Adult bee population density consisted of a visual inspection of each comb and adult bee coverage estimated to the nearest 0.5 frame coverage (1 = fully covered in adult bees) [43]. The *Varroa* infestation rate was determined using fine-powder confectioners’ sugar method [44]. Five treatments (n = 10/treatment) consisted of untreated cardboard impregnated with liquid paraffin (negative control), 1% and 5% (v/v) of cardamon (acaricidal activity was shown in previous study) [30], plasma-treated cardboard impregnated with 5% (v/v) of cardamon essential oil and formic acid 65% (v/v) (positive control) (Mite Away Quick Strips, NOD Apiary Products). Atmospheric-pressure plasma condition was applied at the argon flow rate of 0.50 Lpm for 30 second with a working distance of 1.0 cm. The treatment areas of the plasma jet on the surface of cardboard piece are shown in S3 Fig. Plasma treatment was performed of both sides of the cardboard piece. The treatments were assigned using a stratified random design in which colonies were ranked, high to low mite infestation, and treatments assigned down the rank in groups of ten, to ensure balanced mite infestations across treatment groups. Two strips (2.5 cm × 22 cm) of each treatment were applied weekly for four weeks. Formic acid was treated at 1 and 3 weeks of experiment following the manufacturer’s instructions, two pads per hive total. Colonies were evaluated for adult bee population density and mite infestation at day 21 and 35 of experiment. In the second experiment, 28 colonies with 6–10 frames of bees from the 1^st^ experiment were chosen to test with four treatments in October 2021. Treatments (n = 7/treatment) consisted of cardboard pieces impregnated with mixed essential oils of 2.5% (v/v) clove and 2.5% (v/v) cardamon, plasma-treated cardboard pieces impregnated with 5% (v/v) of clove, plasma-treated cardboard piece impregnated with 5% (v/v) of cardamon and untreated (control) colony were assigned as treatment. Two strips (2.5 cm × 22 cm) of each treatment were put in hives weekly for three weeks. Mite infestation and adult bees were counted before treatment and 28 days after the beginning of the treatment. The efficacy of acaricides was calculated according to the Henderson and Tilton formula [45].

### Statistical analysis

The JMP software version 11.2 for Mac (SAS Institute Inc.) was used to analyze the data. Normality of data was checked using the Shapiro-Wilk test. One-way ANOVA followed by Tukey-HSD analysis was performed when data was normally distributed. The non-parametric Kruskal-Wallis test followed by a Steel-Dwass posthoc multiple comparison was applied to determine if data was not normally distributed. The p-values < 0.05 were considered significant.

## Results

### Plasma characterization and optical emission spectra

The typical voltage and current waveforms of the atmospheric-pressure-argon-plasma jet was measured. The amplitude of the average voltage was 4.758 kV and an intermediate frequency was 831 kHz (S4 Fig). Reactive species intensity produced by plasma is represented in S5 Fig. Different species of Reactive Oxygen Species (ROS) and Reactive Nitrogen Species (RNS) were determined on the spectrum. Excited atoms of O_3_, OH, N_2_ and N_2_^+^ were high-intensity detected. The intensity peaks of reactive species were found to be higher when the gas flow rate was increased from 0.25 to 1.0 L/min. As the gas flow rate increases, the higher of reactive species intensity was detected. The relative intensity of O_3_, OH, N_2_ and N_2_^+^ at gas flow rate 1.0 L/min were 12,495 (a.u.), 14,099 (a.u.), 8,762–2,040 (a.u.) and 707 (a.u.), respectively (S5a Fig). The relative intensity of reactive species was lower when gas flow rate at 0.50 L/min applied (O_3_ = 3,425 (a.u.); OH = 3,860 (a.u.); N_2_ = 1,487–6,240 (a.u.) and N_2_^+^ = 558 (a.u)) (S5b Fig). Approximately, 32-fold decrease of O_3_ and OH intensities was obtained at gas flow rate 0.25 L/min (S5c Fig).

### Suitable solvent for essential oil

**Fig 2:**
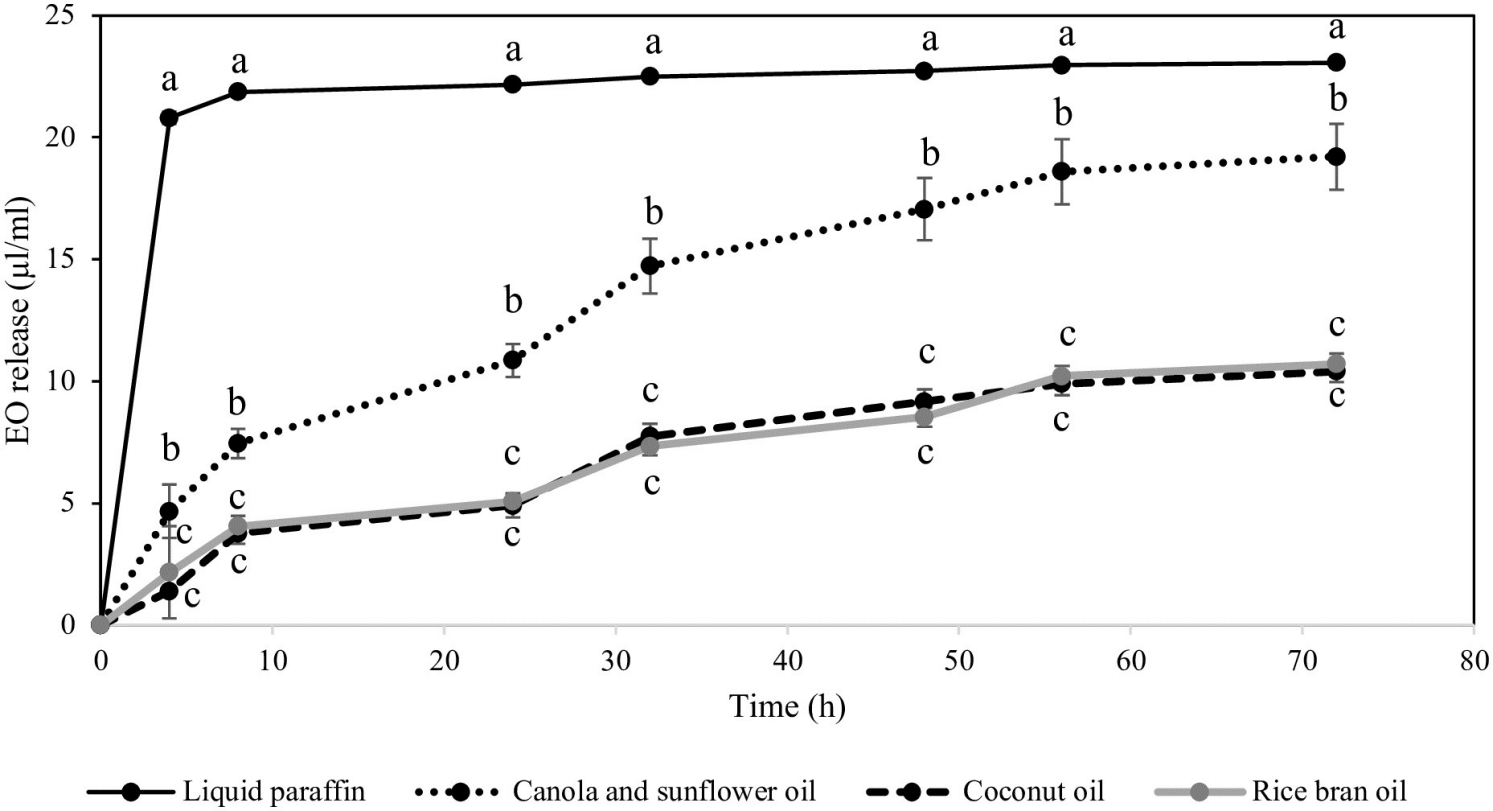
Release rate (mean ± SE) of cinnamon essential oil from cardboard pieces using different solvent, liquid paraffin, canola and sunflower oil, coconut oil and rice bran oil (n = 6/treatment group). Different letters indicate statistically significant differences (Kruskal-Wallis test, Steel-Dwass posthoc test; p < 0.05). Comparisons were made between solvents at each time interval.

The release capacity of cinnamon oil in four different solvents is shown in Fig 2 and S1 Table. Essential oil had the highest release in liquid paraffin followed by canola and sunflower, coconut and rice bran oils at each time intervals (Kruskal-Wallis test, p < 0.05). Cinnamon was dramatically released in liquid paraffin after 4 h of incubation (20.8035 ± 0.2665 μl/ml) and slow released until reaching a plateau, with the release rate of 23.0540 ± 0.0919 μl/ml at 72 h. The release of essential oil in canola and sunflower oil was gradual increased with value of 19.2013 ± 1.3398 μl/ml at 72 h. The lowest release of cinnamon was observed in coconut and rice bran oils.

### Essential oils absorption capacity of cardboard pieces

**Fig 3:**
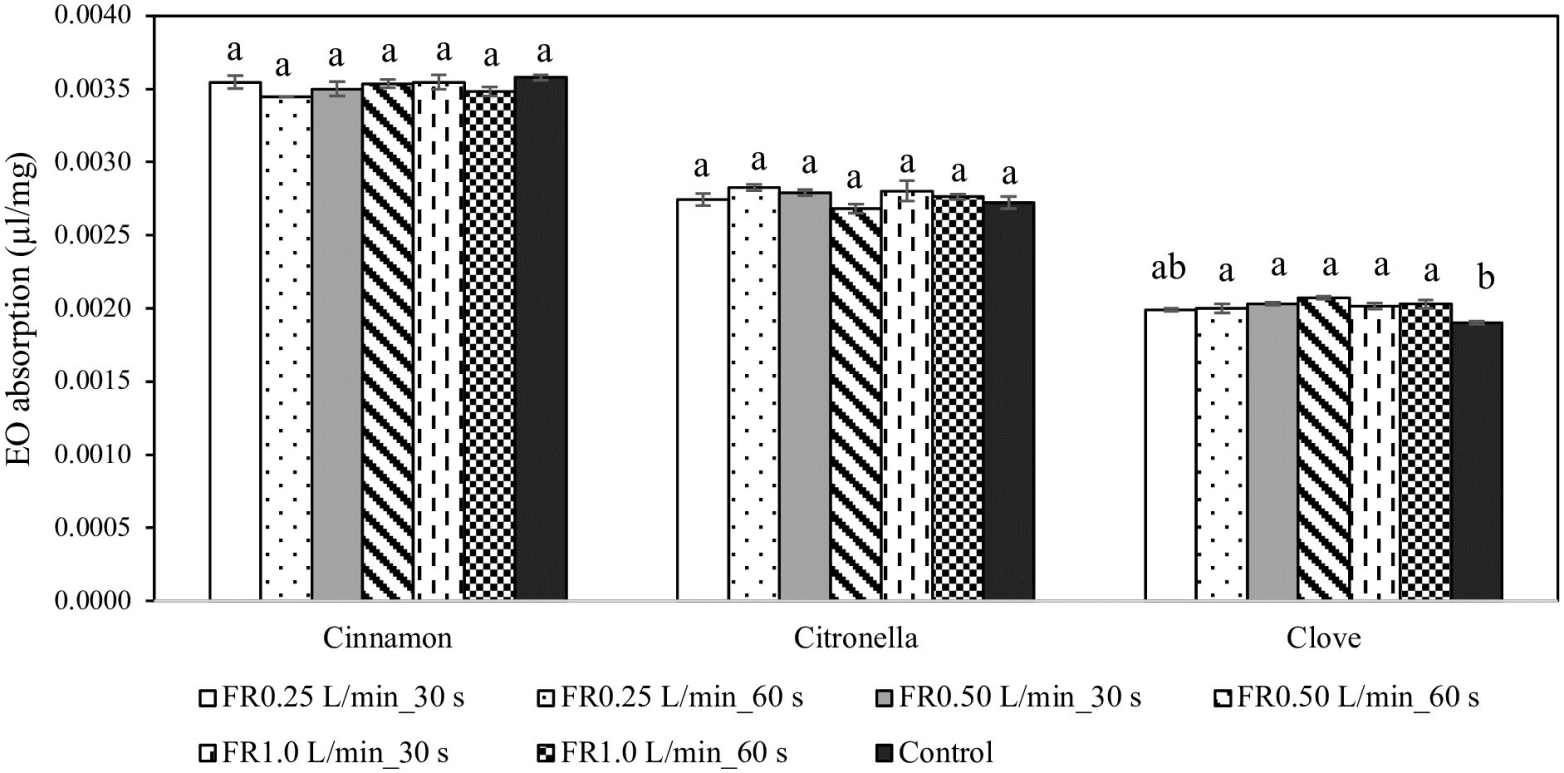
Absorption capacity (mean ± SE) of cinnamon, citronella and clove essential oils by argon plasma-treated cardboard pieces at different gas flow rate and duration of treatment (n = 3/treatment group). Different letters indicate statistically significant differences (ANOVA, Tukey-HSD; p < 0.05).

Three essential oils, cinnamon, citronella, and clove were absorbed by cardboard pieces at differing rates (Fig 3). The absorption capacity was cinnamon > citronella> clove. The amount of absorbed essential oils was about 0.00354 – 0.00358 μl/mg, 0.00268 – 0.00282 μl/mg and 0.00190 – 0.00208 μl/mg for cinnamon, citronella, and clove, respectively. The results showed that argon plasma at different flow rates and time did not affect the essential oil absorption of cardboard piece for cinnamon (ANOVA: F = 1.5904, DF = 6, 14, p = 0.2219) and citronella (ANOVA: F = 1.7147, DF = 6, 14, p = 0.19). However, absorbed clove was significantly enhanced by the plasma treatment of cardboard piece (ANOVA: F = 8.0166, DF = 6, 14, p = 0.0007).

### Characteristic of essential oils release of cardboard pieces

**Fig 4:**
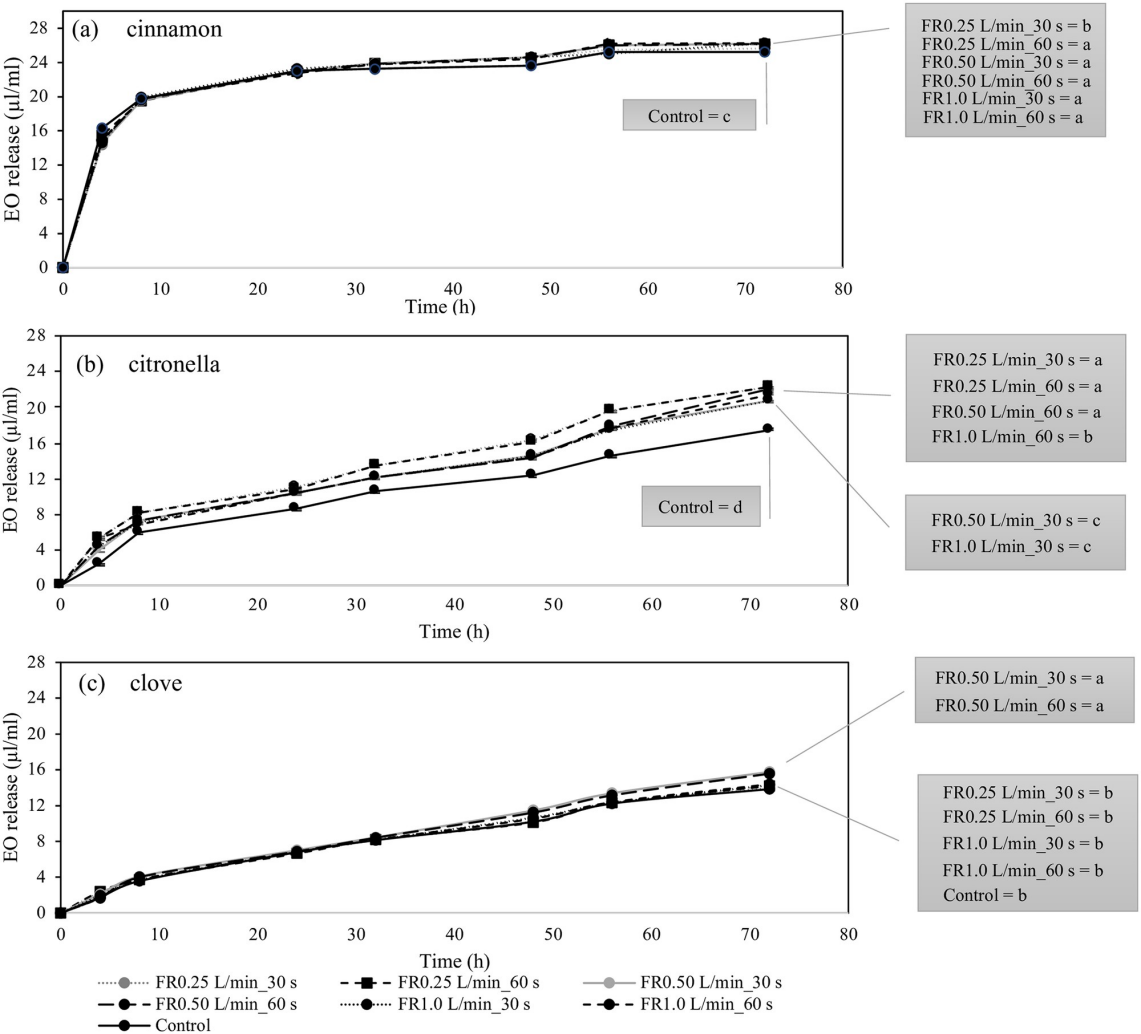
Release rate (mean ± SE) of cinnamon (a), citronella (b) and clove (c) essential oils by argon plasma-treated cardboard pieces at different gas flow rate and duration of treatment (n = 3/treatment group). Different letters indicate statistically significant differences (Kruskal-Wallis test, p < 0.05 followed by a Steel-Dwass posthoc multiple comparisons). Comparisons were made between treatment at each time interval.

The release rate of essential oils by cardboard pieces was very different. Cinnamon was dramatically released at 0–4 h of incubation and plasma-untreated cardboard pieces had the highest release rate (16.3022 ± 0.0887 μl/ml) at this time (Kruskal Wallis test, p = 0.0226) (Fig 4 and S2 Table). The cinnamon oil release rate of atmospheric plasma-treated cardboard pieces was significantly higher at 8 h until 72 h of incubation. At the end of incubation, the release of cinnamon was lowest in the control (untreated cardboard pieces) (25.2447 ± 0.0476 μl/ml) (Fig 4a). The release rate of citronella from cardboard pieces was significantly increased after plasma treatment at all time periods (Fig 4b and S2 Table). The highest release was observed in cardboard pieces treated with argon plasma at flow rate of 0.25 L/min for 30 and 60 s (22.2418 ± 0.0683 and 22.3398 ± 0.0439 μl/ml, respectively) and 0.50 L/min for 60 s (22.0493 ± 0.0962 μl/ml) at the end of incubation. Argon plasma also increased the release rate of clove oil, especially, at the gas flow rate of 0.50 L/min for 30 and 60 s (Fig 4c and S2 Table). Clove was released about 15.5685 – 15.7733 μl/ml for both treatments and 13.8336 ± 0.0591 for untreated cardboard pieces at 72 h of incubation (S2 Table).

### Evaporation of cinnamon essential oil by cardboard pieces

**Fig 5:**
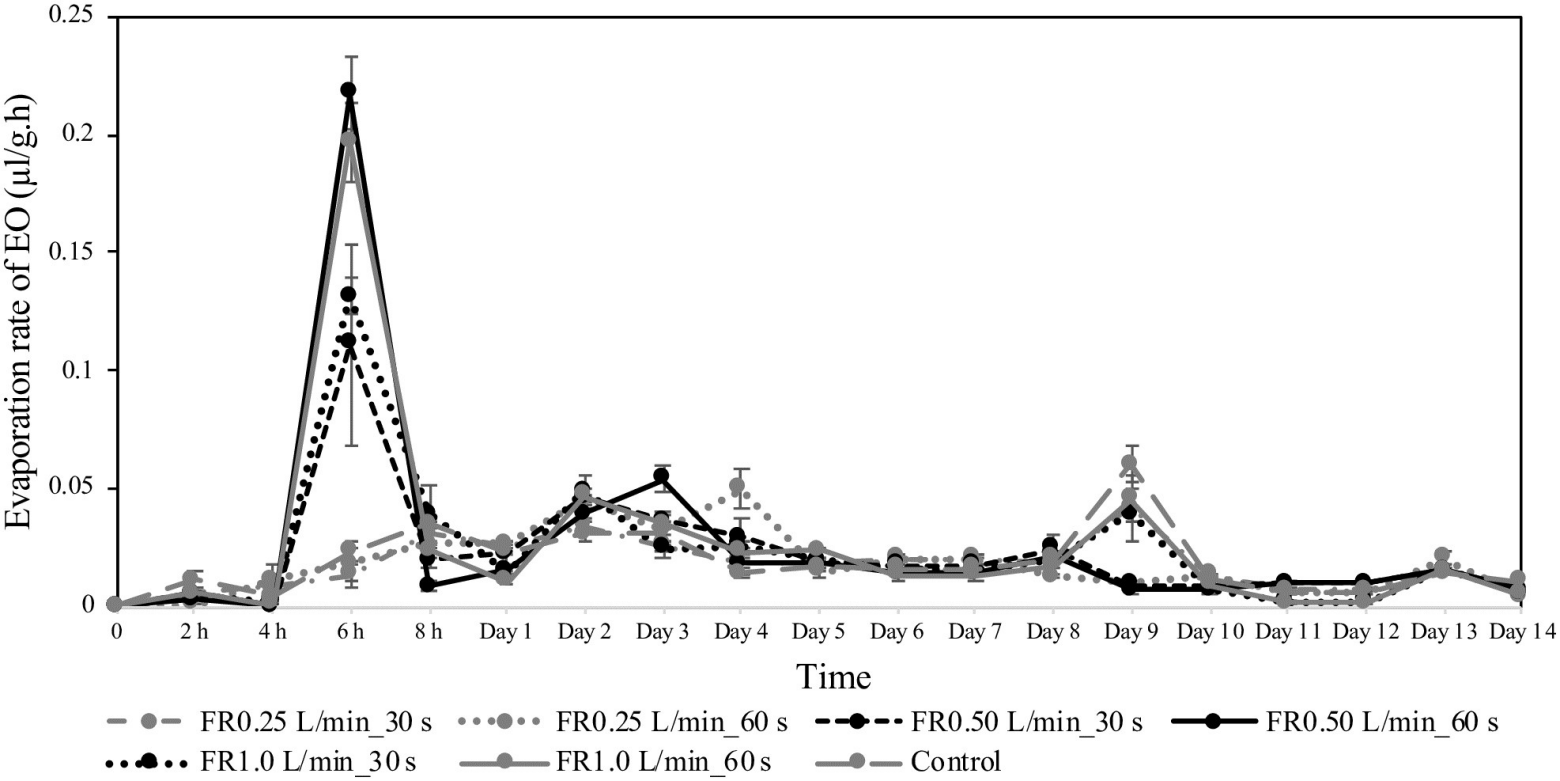
Evaporation rate (mean ± SE) of cinnamon essential oil from cardboard pieces treated with argon plasma jet at different gas flow rate and duration of treatment (n = 3/treatment group). Different letters indicate statistically significant differences (Kruskal-Wallis test, p < 0.05 followed by a Steel-Dwass posthoc multiple comparisons). Comparisons were made between treatment at each time interval.

The evaporation rate of cinnamon oil from cardboard pieces over 14 days is presented in Fig 5 and S3 Table. The highest evaporation of cinnamon was observed at 6 h for all treatments (the statistical analyses can be found in S3 Table). The plasma conditions used in this study seemed to affect the cardboard property for essential oil evaporation for 5 days. The results demonstrated that cinnamon was significantly evaporated from argon plasma-treated cardboard pieces at the flow rate of 0.50 L/min for 60 s (0.2175 ± 0.0148 μl/g.h) and 1.0 L/min for 30 and 60 s (0.1314 ± 0.0076 and 0.1970 ± 0.0166 μl/g.h, respectively). The evaporation rate was then rapidly decreased at 8 h and slow reduced until the end of experiment.

### Plasma-treated cardboard pieces impregnated with essential oils to control *Varroa* mites under field conditions

**Fig 6:**
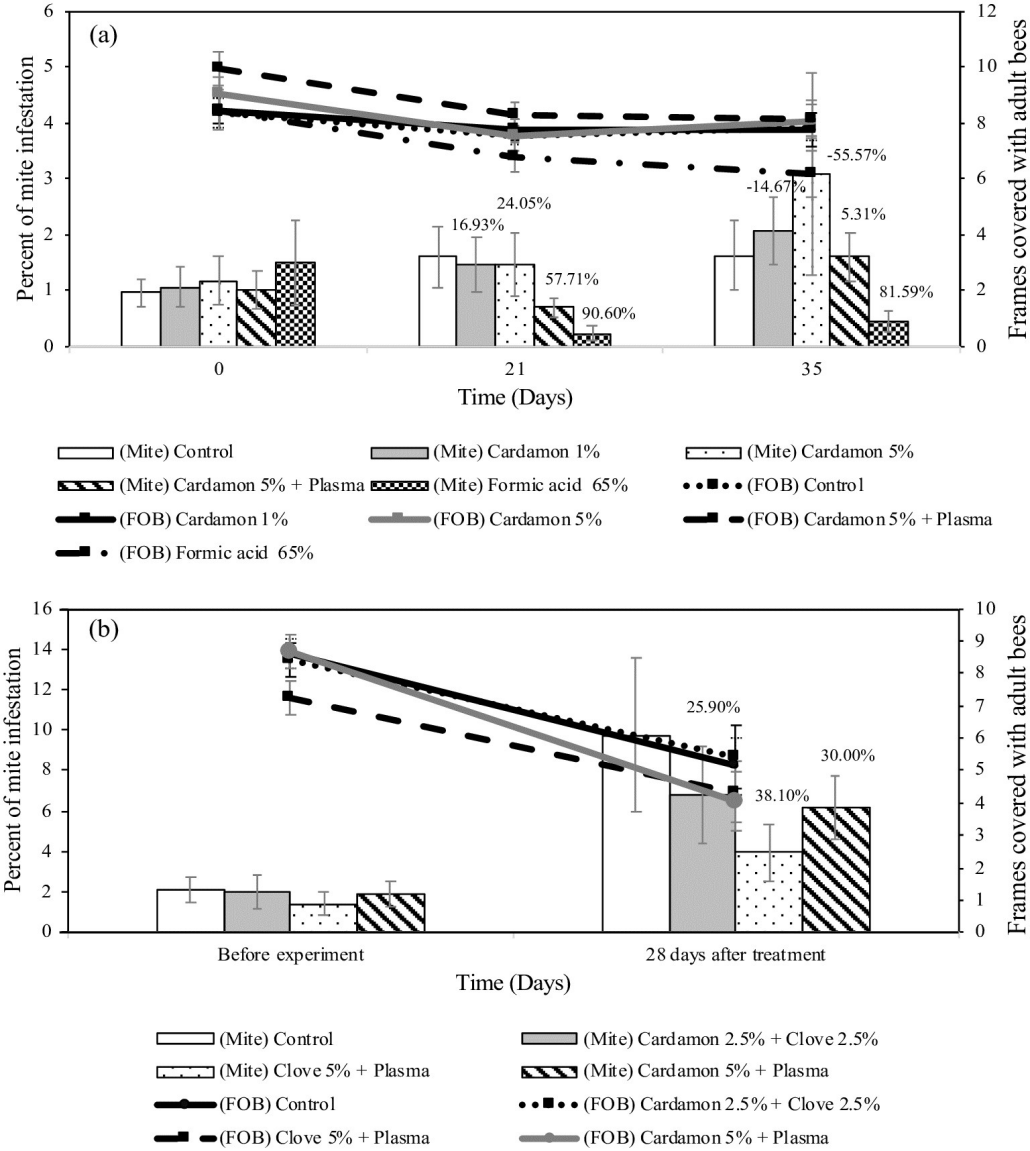
Effect of treatments on *Varroa* infestation rate and adult bee population under field conditions. Average number (mean ± SE) of Varroa mite infestation (columns) and average number of frames covered with adult bees (lines) in colonies after treated with plasma untreated and treated-cardboard pieces impregnated with different concentration of cardamon oil (a) and mixed essential oils between cardamon and clove oil (b). Comparisons were made at each time interval (Kruskal-Wallis test, p<0.05). Efficiency of treatments (%) was also reported (above each column).

The results of the first experiment demonstrated that formic acid (65% v/v) showed high efficiency to control *Varroa* mite (90.60% and 81.59% at day 21 and 35, respectively) (Fig 6a). Plasma-treated cardboard pieces impregnated with 5% cardamon was more efficient in reducing *Varroa* than untreated cardboard pieces soaked with 1% or 5% cardamon, which resulted in a 57.71% and 5.31% reduction of *Varroa* at day 21 and 35 of treatment respectively. One dead colony was observed in the formic acid treatment. Mite population in all treatments increased at day 35 of treatment. Adult bees were slightly decreased for all treatments (Fig 6a). At the end of the experiment, a 26.63% bee decline was observed in colonies treated with formic acid (6.20 ± 0.83 frames of bees) and 18.09% after treated with plasma-treated cardboard pieces impregnated with 5% of cardamon (8.15 ± 0.64 frames of bees) (S4 Table). While control (7.85 ± 0.49 frames of bees) and untreated cardboard soaked with 1% and 5% cardamon had about 5.99–11.05% bee losses (7.75 ± 0.58 and 8.05 ± 0.60 frames of bees) (S4 Table). In the second experiment, four colonies died at the end of experiment (1 of control, 1 colony of 5% clove and 2 colonies of 5% cardamon treatments). The highest efficacy obtained was with plasma-treated cardboard pieces soaked with 5% (v/v) clove oil (38.10%) (Fig 6b). The reduction rate of *Varroa* were 25.90% after treated with cardboard piece impregnated with mixed essential oils of 2.5% (v/v) clove and 2.5% (v/v) cardamon and 30.01% of plasma-treated cardboard piece impregnated with 5% (v/v) cardamon (Fig 6b and S5 Table). The infestation of *Varroa* in control coloniesincreased from 2.12 ± 0.63% to 9.75 ± 3.79% at day 28 (Fig 6b). The infestation rate of *Varroa* in essential oil treatments was also elevated about 2.84–3.40-fold (6.80 ± 2.36%, 3.94 ± 1.43% and 6.17 ± 1.58% in untreated cardboard piece soaked with mixed 2.5% clove and 2.5% cardamon, plasma-treated cardboard piece impregnated with 5% clove and 5% cardamon, respectively (S5 Table). The decline of adult bee populations were 40.58% (FOB = 5.12 ± 0.87), 35.71% (FOB = 5.40 ± 0.97), 40.23% (FOB = 4.33 ± 0.95) and 53.21% (FOB = 4.06 ± 0.89) in control, untreated cardboard piece soaked with mixed 2.5% clove and 2.5% cardamon, plasma-treated cardboard piece impregnated with 5% clove and 5% cardamon, respectively by the end of experiment (Fig 6b, S5 Table).

## Discussion

Essential oils, cinnamon, citronella and clove have been shown high acaricidal activity on *Varroa* mites under laboratory conditions [27, 30, 46]. However, the results of using essential oils under colony conditions are not always consistent [27, 47]. Numerous factors such as climate, temperature, humidity and behavior of honeybees could affect the evaporation rate of essential oils. Therefore, developing the materials for long-lasting acaricidal activity in the hive, concentration of essential oil and type of essential oil dissolving solvent are important factors for their application in hives. Our results revealed that higher release patterns of cinnamon oil was observed in liquid paraffin and the rate of release was higher during the first four hours. This may be due to diffusion through the solvent in the first hours. Solubility parameters can also be associated with the release rate of essential oils in solvent [48, 49]. The diffusion and solubility may have occurred due to the content of constituents in both of essential oils and solvents [50]. Therefore, the analysis of solubility, kinetics of the sorption and diffusivity of essential oils and solvents can give useful information in the development of new miticide products for *Varroa* control. In this study, the atmospheric pressure plasma was used as a tool to modify the surface of cardboard for improving the efficacy of essential oils for *Varroa* mites control. Among three different essential oils, cinnamon was more highly absorbed by cardboard pieces followed by citronella and clove. However, argon plasma conditions used in this study did not significant change the cardboard piece surface for cinnamon and citronella absorption. Conversely, clove oil absorption by cardboard pieces was increased after plasma treatment. This might be due to the absorption of cinnamon and citronella closely reached the maximum loading capacity of cardboard piece. However, a fractured and scaly surface of cardboard after plasma treatment [40] could be attributed to the enhance of clove absorption. Additionally, composition of essential oil, its solubility and material properties might play a key role for the absorption capacity of essential oils [51]. The release pattern of three essential oils was also different and the release rate was increased after plasma treatment. This could be attributed to the surface modification by plasma that allows essential oil components from cardboard pieces to diffuse into liquid paraffin. In the field experiments, acaricidal activity of cardamon and clove essential oils delivered using plasma-treated cardboard pieces was investigated. All treatments reduced the mite population after 21 days of experiment. Formic acid (65%) had the highest efficacy (90.60%) to control *Varroa*. The efficacy of cardamon oil delivered using plasma-treated cardboard pieces (57.71%) was two times higher than that of cardamon oil delivered using untreated cardboard pieces (24.05%) in the first experiment. Also, plasma-treated cardboard pieces impregnated with 5% of each clove and cardamon oils had about 30.01–38.10% of mite reduction which greater than untreated cardboards soaked with mixed 2.5% of clove and cardamon essential oils. However, the efficacy of acaricidal activity was not significant different between treatments in both the first and second experiments. A decline in adult bees was observed during these experiments, especially it was higher in the colonies treated with formic acid (26.63%). Formic acid can increase the mortality of brood and adult bees [52]. Several factors such as ambient temperature, colony size, brood and bee populations, concentration and dispenser could effect the efficacy of essential oils to control *Varroa* mite. Therefore, the relative of these factors to each other, should be further investigated. The results suggest that atmospheric pressure plasma may affect some properties of the cardboard surface and could improve the continuous or slow release of essential oils. Although, essential oils demonstrated lower ability to kill *Varroa* mite in these studies when compared to formic acid, the results still provide insight into using plasma technology to improve the honeybee mites control. Boonmee et al. reported that atmospheric pressure plasma did not significantly change the surface properties of cardboard piece [40]. However, impregnation of plasma-treated cardboard pieces in 5% (v/v) of cardamon essential oil can decrease *Tropilaelaps* mite under the field conditions [40]. Several essential oils have demonstrated acaricidal activity [30, 32, 46, 53, 54] but some essential oils negatively affect honeybees. This might be the reason that only a few of them have been tested under colony conditions [33, 40, 55, 56]. Several studies have been previously reported that clove oil had high efficacy for *Varroa* controlling both in laboratory and in the field conditions [30, 46, 57, 58]. Oregano essential oil has demonstrated acaricidal activity on *Varroa* with efficiency of 57–74% depending on the concentration used [59]. Efficacy of clove and oregano essential oils with different delivery methods to control *V*. *destructor* has been reported [55]. Oregano oil delivered using electric vaporizers showed high varroacidal efficacy (97.4 ± 0.68%) since vaporizers could provide a more continuous evaporation of volatile compounds [55]. Many factors need further study, including the essential oil delivery method, for an effective and safe continuous release for mite control. Plasma technology could be a useful technique to improve the essential oils efficiency and /or material properties for the control of honeybee mites. However, the cost and practically of using atmospheric pressure plasma for surface modification of materials must also be considered.

## Supporting information

S1 Table Release rates of cinnamon essential oil using different solvent. (XLSX)

S2 Table Release rates of essential oils. (XLSX)

S3 Table Evaporation rates of cinnamon essential oil. (XLSX)

S4 Table *Varroa* mite infestation in colonies of the first experiment. (XLSX)

S5 Table *Varroa* mite infestation in colonies of the second experiment. (XLSX)

S1 Fig Physical characteristics of cardboard. (PDF)

S2 Fig Experimental honeybee colonies and the essential oils delivered via cardboard pieces application. (PDF)

S3 Fig Atmospheric-pressure plasma treatment on the surface of cardboard piece. (PDF)

S4 Fig Typical waveforms of the discharge current and voltage. (PDF)

S5 Fig Typical optical emission spectrum (OES) of argon plasma jet. (PDF)
