# Supplementary figures and images for: Properties of essential oils absorbed on the surface of cardboard pieces after using atmospheric-pressure plasma treatments to develop long-lasting Varroa miticides in honeybees (Apis mellifera)
Source: PLoS One. 2024 Feb 8;19(2):e0297980. doi: 10.1371/journal.pone.0297980 (PMC10852235; doi:10.1371/journal.pone.0297980)

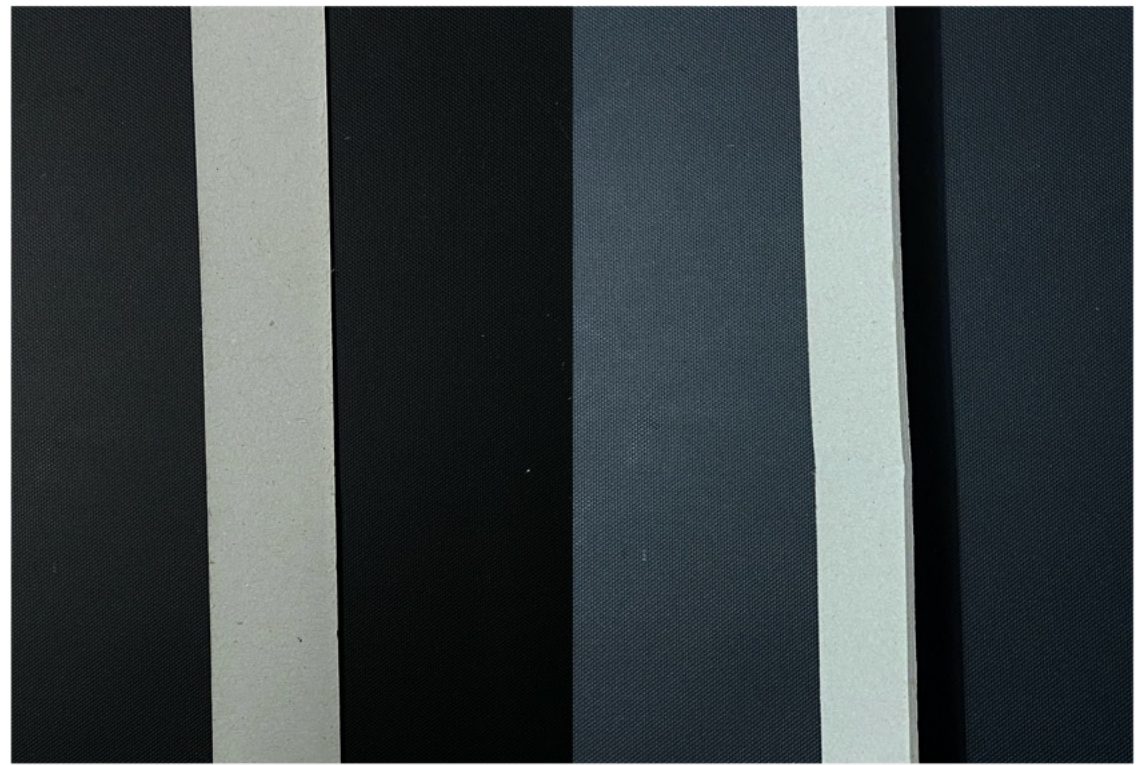

**S1 Figure.** Physical characteristics of cardboard (grey board) used in this study.

Supplement: S1 Fig — (PDF) [file pone.0297980.s006.pdf]

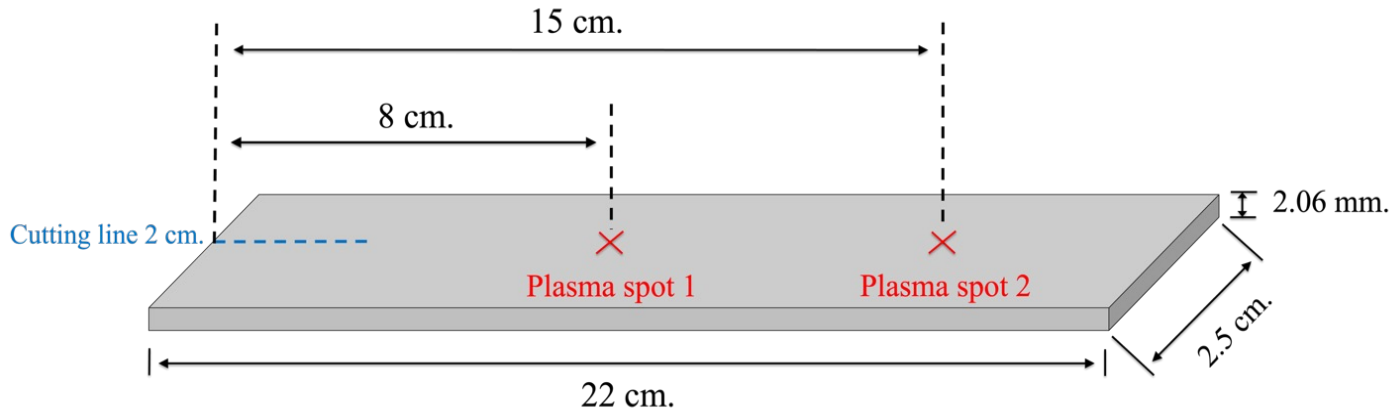

**S3 Figure.** Atmospheric-pressure plasma treatment on the surface of cardboard piece

Supplement: S3 Fig — (PDF) [file pone.0297980.s008.pdf]

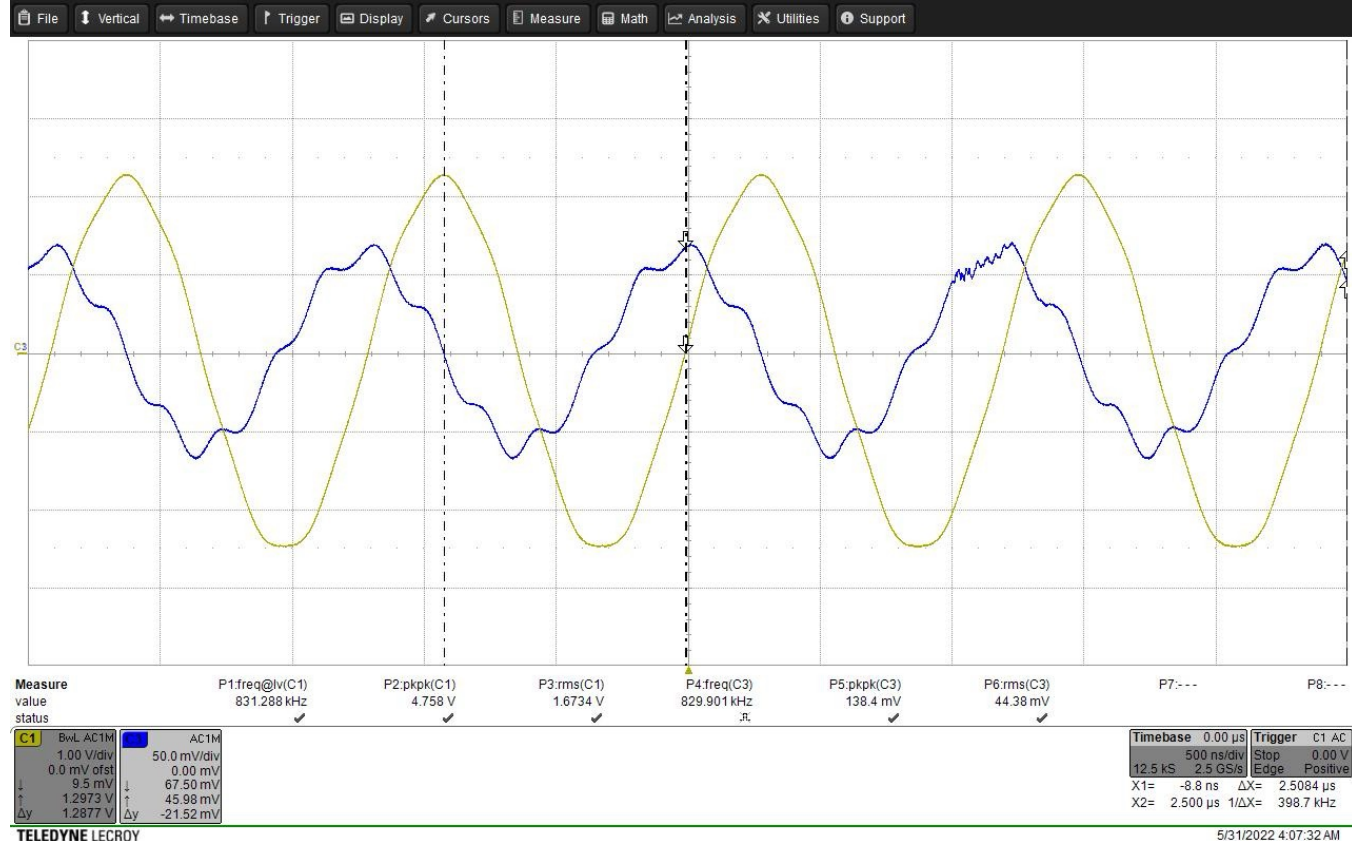

**S4 Figure.** Typical waveforms of the discharge current and voltage.

Supplement: S4 Fig — (PDF) [file pone.0297980.s009.pdf]
