# Supplementary material for: Properties of essential oils absorbed on the surface of cardboard pieces after using atmospheric-pressure plasma treatments to develop long-lasting Varroa miticides in honeybees (Apis mellifera)
Source: PLoS One. 2024 Feb 8;19(2):e0297980. doi: 10.1371/journal.pone.0297980 (PMC10852235; doi:10.1371/journal.pone.0297980)
Supplement: S2 Fig — (PDF) [file pone.0297980.s007.pdf]

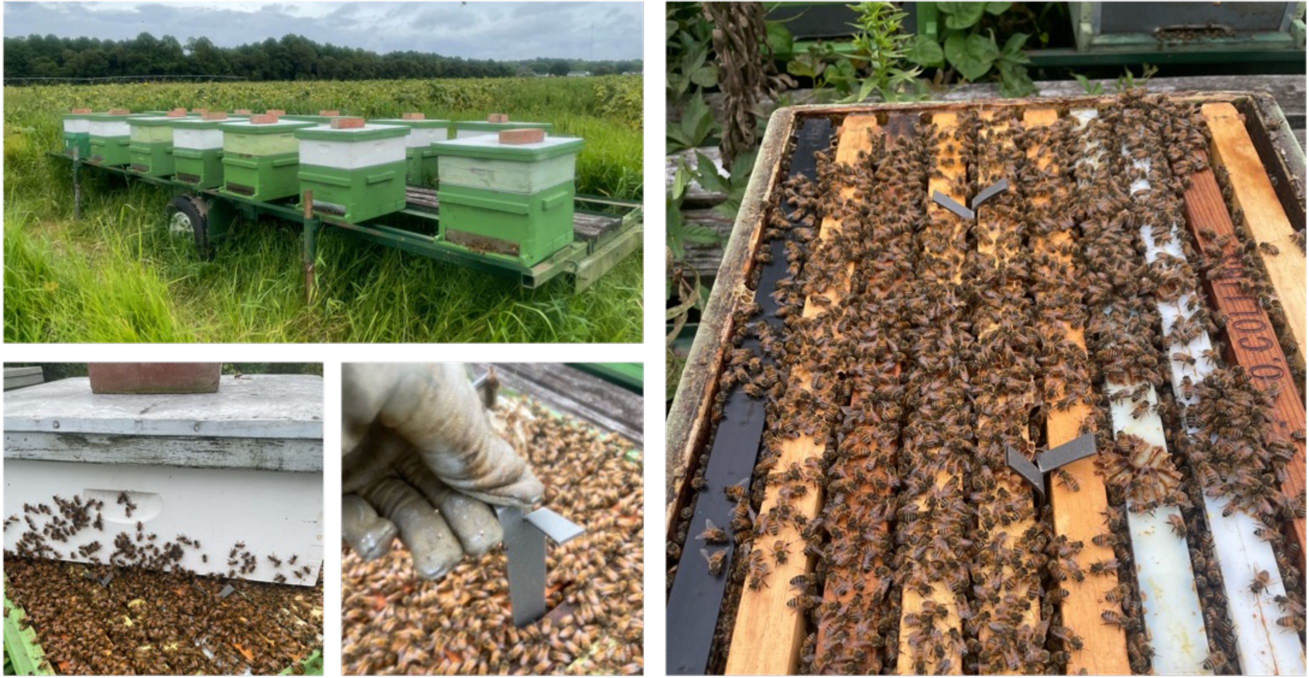

S2 Figure. Experimental honeybee colonies and the essential oils delivered via cardboard pieces application.
