# Supplementary material for: Properties of essential oils absorbed on the surface of cardboard pieces after using atmospheric-pressure plasma treatments to develop long-lasting Varroa miticides in honeybees (Apis mellifera)
Source: PLoS One. 2024 Feb 8;19(2):e0297980. doi: 10.1371/journal.pone.0297980 (PMC10852235; doi:10.1371/journal.pone.0297980)
Supplement: S5 Fig — (PDF) [file pone.0297980.s010.pdf]

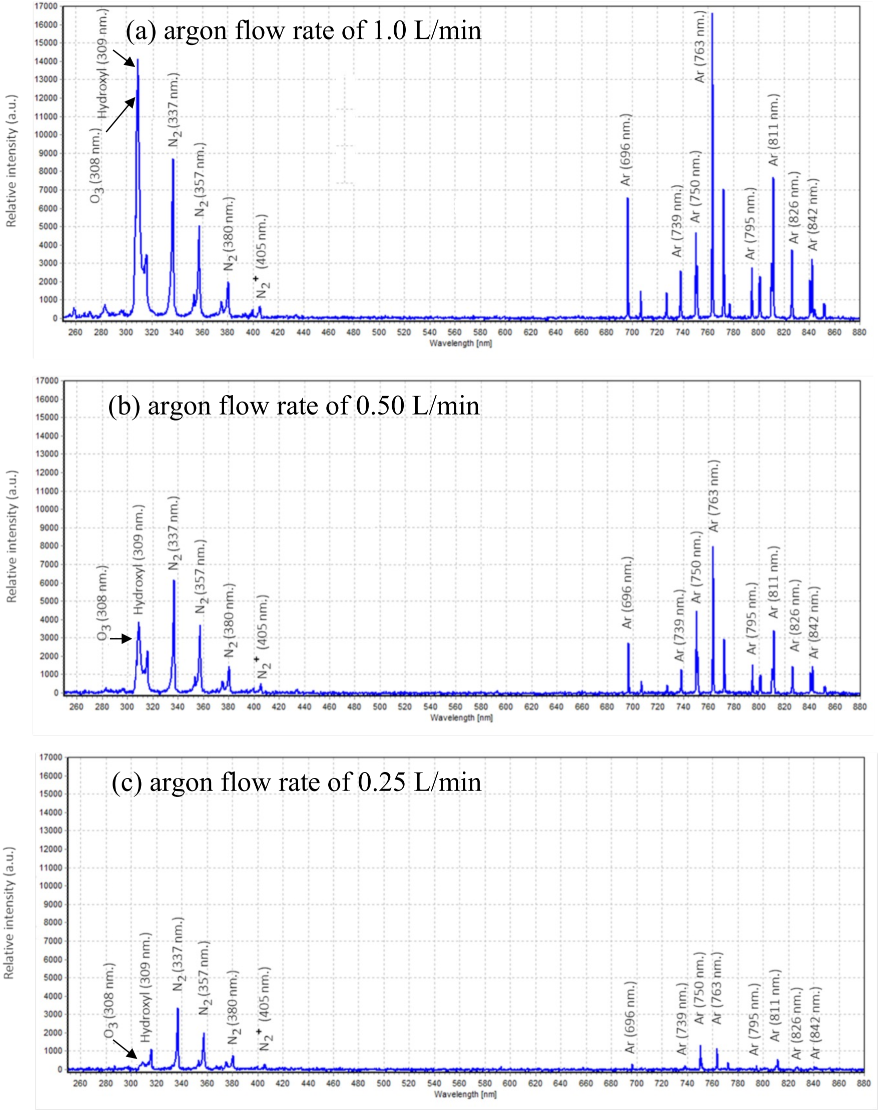

S5 Figure. Typical optical emission spectrum (OES) of argon plasma jet at gas flow rate of 1.0 L/min (a), 0.5 L/min (b) and 0.25 L/min (c).
